# Neutralizing Antibody Screening Using NanoBiT-Based Virus-like Particles of Foot-and-Mouth Disease Type Asia1 Enhances Biosafety and Sensitivity

**DOI:** 10.3390/v17030337

**Published:** 2025-02-27

**Authors:** Hyejin Kim, Dong-Wan Kim, Giyoun Cho, Ji-Hyeon Hwang, Yeonrae Chae, Taejun Kim, Jae Young Kim, Young-Joon Ko, Jong-Hyeon Park, Yoon-Hee Lee, Sung-Han Park

**Affiliations:** Center for Foot-and-Mouth Disease Vaccine Research, Animal and Plant Quarantine Agency, 177, Hyeo-ksin 8-ro, Gimcheon-si 39660, Gyeongsangbuk-do, Republic of Korea; hyejin86@korea.kr (H.K.); dongwan12@korea.kr (D.-W.K.); jihyeonh87@korea.kr (J.-H.H.); codusfo12@korea.kr (Y.C.); taejun0526@korea.kr (T.K.); ivorikim@korea.kr (J.Y.K.); koyoungjoon@korea.kr (Y.-J.K.); parkjhvet@korea.kr (J.-H.P.)

**Keywords:** foot-and-mouth disease, virus-like particles, NanoBiT assay, HiBiT, LgBiT

## Abstract

Background/Objectives: Foot-and-mouth disease (FMD) is a highly contagious class 1 animal disease that affects cloven-hoofed animals, such as cattle, pigs, and goats. Diagnosis and research on live FMD virus (FMDV) typically require biosafety level 3 facilities, which are challenging to maintain due to strict protocols and high costs. The development of NanoBiT-based assays has accelerated in response to the coronavirus disease pandemic, providing safer alternatives for viral research, and is now applicable for general laboratories. This study aimed to develop a NanoBiT-based virus-like particle (VLP) assay for the rapid and safe screening of neutralizing antibodies against FMDV Asia1 Shamir (AS). Methods: We developed an AS VLP with an inserted HiBiT tag that enabled the detection of entry into LgBiT cells through luminescence signals. Results: HiBiT-tagged AS VLPs mixed with anti-serum and introduced into LgBiT-expressing cells led to a reduction in luciferase activity. Therefore, we established a NanoBiT-based viral neutralizing antibody test (VNT) that demonstrated a high correlation (R^2^ = 0.881) with the traditional gold standard VNT. Conclusions: The assay demonstrated high sensitivity and could be performed in BL-2 facilities, offering a safer and more efficient alternative to traditional assays while reducing the need to handle live viruses in high-containment facilities. This method provides a valuable tool for rapid screening of neutralizing antibodies and can be adapted for broader applications in FMDV research.

## 1. Introduction

Foot-and-mouth disease (FMD) is a highly contagious disease in cloven-hoofed animals, including cattle, pigs, and goats [1]. FMD virus (FMDV) belongs to the genus *Aphthovirus* of the family *Picornaviridae* and has a single-stranded, plus-sense RNA genome of approximately 8.5 kb that is translated into a polyprotein. It is subsequently cleaved into four structural proteins (SPs), the P1 region (VP0; VP4 and VP2, VP3, and VP1), to form an icosahedral capsid, and nonstructural proteins (NSPs: L, 2A, 2 B, 2C, 3A, 3 B, 3C, and 3D) [2,3]. The virus exists in seven distinct serotypes (A, O, C, Asia 1, SAT 1, SAT 2, and SAT 3), each with multiple subtypes, none of which confer cross-protection among the different serotypes, complicating FMD control [4,5,6].

Serological tests for FMD detect antibodies (Abs) against viral SPs and NSPs to determine their prevalence and distribution in exposed animals and to monitor the immunity conferred by vaccination in the field [7]. SP tests comprise three methods: viral neutralization test (VNT), solid-phased competition ELISA and liquid-phase blocking ELISA, which are highly sensitive, providing that the virus or antigen used in the test closely matches the strain circulating in the field [8]. VNT and FMDV production require cell culture facilities and live viruses. In addition, it takes 2–3 days to provide results in a biosafety level 3 (BL-3) facility with an appropriate level of biocontainment, as determined by risk analysis [9]. Therefore, a simple, rapid, and easy-to-perform neutralizing antibody (NAb) screening test is urgently needed for use in BL-2 facilities.

Bioluminescent reporters, such as nano-luciferase (Nano-Luc) binary technology (NanoBiT), can be easily detected and quantified. NanoBiT is a two-fragment system consisting of a large N-terminal fragment (LgBiT; 18 kDa) and a small C-terminal fragment (HiBiT; 1.3 kDa, 11 amino acids), which can detect protein–protein interactions within live cells [10,11]. The HiBiT-tagged virus serves as a valuable tool for monitoring viral infections and is widely used for basic and applied viral studies, antiviral screening, and vaccine evaluation [12,13,14]. Recently, we developed a live HiBiT-tagged FMDV Asia1 Shamir (AS) in a BL-3 facility and confirmed that inactivated HiBiT-tagged FMDV can detect seroconversion in the anti-sera of guinea pigs within a BL-2 facility [15]. The AS-HiBiT VLP-based NanoBiT assay demonstrates strong potential for clinical and experimental applications by enabling sensitive NAb detection. The insertion site, serine 153 (153S) in VP1, was chosen due to its location within the GH-loop, a structurally flexible region known to tolerate genetic modifications while maintaining viral stability and antigenicity [16]. Additionally, 153S is adjacent to the RGD motif, which is crucial for integrin-mediated cellular entry of FMDV. This positioning ensures that HiBiT insertion does not disrupt receptor binding, allowing real-time tracking of virus-like particle (VLP) interactions while preserving functional properties. This approach builds upon previous applications of NanoBiT technology in virology, where luminescent tagging has been widely employed for real-time monitoring of viral interactions. NanoBiT research has been applied to coronaviruses, Nipah virus, norovirus, Senecavirus A, and porcine reproductive and respiratory syndrome virus [17,18,19,20,21,22].

Virus-like particles (VLPs) are similar to viral particles and are required for synthetic processing and self-assembly into empty viral capsids, but they do not contain infectious genetic material [23]. FMD VLPs require the expression of the P1-2A precursor to form capsids and the 3C protease that cleaves P1-2A into capsid proteins [23,24]. FMD VLPs have been developed using several expression systems with different host cells, including insect cells, mammalian cells, baculoviruses, and yeast platforms [25,26,27,28,29,30]. VLPs expressed in *E. coli* or the yeast *Hansenula polymorpha* are similar to native FMDV and can effectively trigger immune responses in pigs and mice [31,32].

In this study, we aimed to develop a rapid screening assay to detect NAbs against the FMD Asia1 serotype using split Nano-Luc complementation-based VLPs in live cells. HiBiT-tagged AS VLPs (AS-HiBiT VLPs) were generated in mammalian cells and introduced into LgBiT-expressing cells, producing luminescence signals. Using these principles, we developed a NanoBiT-VNT as a rapid screening assay of NAbs using FMD AS-HiBiT VLPs, which can be performed in BL-2 facilities.

## 2. Materials and Methods

### 2.1. Cells

In this work, the cell lines used were human embryonic kidney (HEK) 293T cells (ATCC CRL-3216), fetal porcine kidney LF-BK cells provided by the Agricultural Research Service (ARS) of the US Department of Agriculture (USDA), and stable LgBiT-expressing LF-BK cells (stable LgBiT cells) [15,33]. The cells were maintained in Dulbecco’s Modified Eagle’s Medium (DMEM; Corning, Union City, NJ, USA) supplemented with 10% inactivated fetal bovine serum (Gibco, Grand Island, NY, USA) and 1% penicillin-streptomycin solution (Corning).

### 2.2. Production of AS-HiBiT VLPs

**Figure 1:**
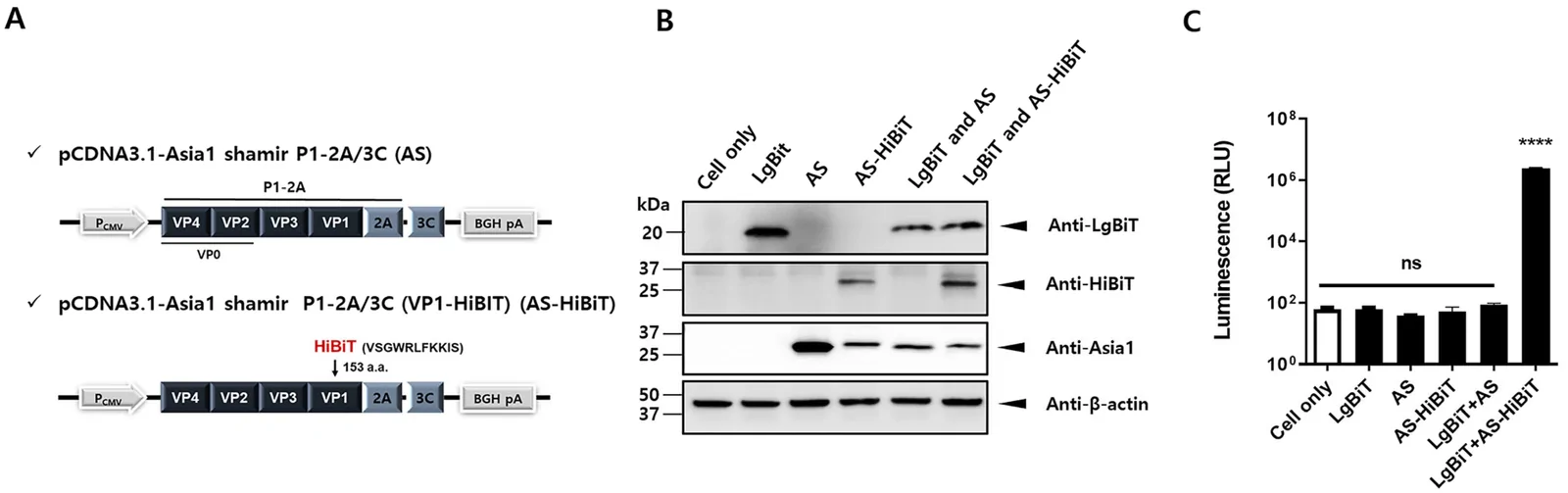
Schematic and validation of HiBiT-tagged Asia1 Shamir (AS-HiBiT) plasmids and luciferase activity for the nano-luciferase binary technology (NanoBiT) assay. (**A**) Schematic representation of the HiBiT-tagged AS virus-like particles (AS-HiBiT VLPs). The AS-HiBiT plasmid includes the HiBiT inserted into the VP1 protein (153S) of the pCDNA3.1-AS P1-2A/3C (AS) plasmid. (**B**) Western blot analysis confirming the expression of LgBiT, HiBiT, and AS proteins of HEK293T cells transfected with the respective plasmids; β-actin was used as an internal control. (**C**) Luminescence assay showing luciferase activity in cells transfected with plasmids. Cells co-transfected with LgBiT and AS-HiBiT plasmids exhibited a significant increase in luminescence compared with cells transfected with LgBiT or AS-HiBiT alone. The results are presented as means ± SEM of triplicate wells. Statistical significance was analyzed using one-way ANOVA followed by Tukey’s multiple comparisons test. ****, *p* < 0.0001; ns, no significant difference.

The AS (GenBank accession No. JF739177) P1-2A/3C gene was cloned into pcDNA3.1, resulting in the plasmid AS P1-2A/3C (AS VLP). To generate HiBiT-tagged FMDV AS-VLP clones, HiBiT tag (VSGWRLFKKIS) flanked by GSSG- and GSG-linkers (GSSGVSGWRLFKKISGSG) were inserted after serine at position 153 (153S) of the VP1 region of AS P1-2A/3C to yield the plasmid AS P1-2A/3C-HiBiT (AS-HiBiT VLP, Figure 1A). The AS or AS-HiBiT VLP plasmids were transfected into HEK293T cells using Fugene HD transfection reagent (Promega, Madison, WI, USA) at a DNA plasmid-to-reagent ratio of 1:3. After incubation for 48 h, the AS or AS-HiBiT VLPs were subjected to freeze-thaw cycles and centrifuged at 3000 × *g* for 10 min at 4 °C to remove cell debris.

### 2.3. Concentration and Purification of AS-HiBiT VLPs

Following our previously reported methods for concentrating inactivated viruses [34], the AS or AS-HiBiT VLPs were treated with 7.5% polyethylene glycol 6000 (Sigma-Aldrich, St. Louis, MO, USA). To purify the VLPs, suspensions were layered on top of a 15–45% (w/v) sucrose density gradient, ultra-centrifuged at 100,000 × *g* for 4 h, collected, and the absorbance of each fraction was measured at 280 nm. The purified VLP suspension was placed on a formvar-coated grid and negatively stained with 1% uranyl acetate. VLPs were examined using transmission electron microscopy (TEM; H-7100FA; Hitachi, Tokyo, Japan).

### 2.4. SDS-PAGE and Western Blot Assay

Target proteins were analyzed via SDS-PAGE with a Pierce Silver Stain kit (Thermo Fisher Scientific Inc., Rockford, IL, USA) and Western blotting. Concentrated proteins underwent silver staining following the manufacturer’s protocol. For Western blotting, proteins were transferred to a PVDF membrane (Bio-Rad Laboratories, Hercules, CA, USA), detected using primary and HRP-conjugated secondary Abs, and visualized with an enhanced chemiluminescence substrate (Amersham, Buckinghamshire, UK) using an Azure C600 device (Azure Biosystems, Dublin, CA, USA). The Abs used included anti-HiBiT (Promega), anti-LgBiT (Promega), anti-rabbit IgG (Millipore, Billerica, MA, USA), anti-mouse IgG (Millipore), and Anti-FMDV Asia 1 (PrioCHECK FMDV Type Asia1 Antibody ELISA kit; Prionics AG, Schlieren-Zurich, Switzerland).

### 2.5. Detection of AS-HiBiT VLP Entry Using a NanoBit Assay

Stable LgBiT cells were seeded (5,000, 10,000, or 20,000 cells/well) into a white 96-well plate and incubated at 37 °C. The following day, the cells were washed with PBS, and a mixture of serum-free DMEM and AS-HiBiT VLPs (in equal proportions; 50 µL) was added to the 96-well plate. The plates were then incubated at 37 °C for 1, 4, 6, or 24 h. After incubation, the cells were washed twice with PBS and replenished (100 µL/well) with PBS, serum-free DMEM((-)FBS DMEM), DMEM supplemented with 2% FBS (2% FBS DMAM) or opti-MEM. Luciferase activity was measured using a Nano-Glo Luciferase Assay System (Promega) according to the manufacturer’s instructions. Viral entry into the cells and background interference from buffers (including media) were assessed based on the luciferase activity levels.

### 2.6. Animal Serumdm

All animal experiments and serum collections were conducted with the approval of the Institutional Animal Care and Use Committee of the Animal and Plant Quarantine Agency (APQA) of the Republic of Korea (IACUC approval No. 2024-860). Serum from guinea pigs (*n* = 4) vaccinated with a commercial vaccine, including AS at a 0.2 mL/dose, was collected at 0 and 28 days post-vaccination (dpv). Serum from two-month-old pigs (*n* = 5) vaccinated in-house with an FMDV AS antigen was collected at 0, 28, 42, and 56 dpv. In addition, blood samples from pigs vaccinated with the monovalent FMDV AS vaccine (*n* = 58) were provided by Dr. Young-Joon Ko of the APQA of the Republic of Korea [35]. A total of 58 serum samples were analyzed in this study. Based on the traditional VNT titers using live virus, the samples were categorized as follows: VN titer < 0.9 (*n* = 17), VN titer 0.9-1.51 (*n* = 17), and VN titer ≥ 1.65 (*n* = 24). These serum samples were used for all subsequent analyses, including the traditional VNT and NanoBiT-VNT assays.

### 2.7. FMDV Asia1 Ab ELISA and Traditional Gold Standard VNT

The sera were heat-inactivated at 56 °C for 30 min and tested using the FMDV Asia1 ELISA (Prionics AG, Schlieren-Zurich, Switzerland) following the manufacturer’s protocol. In serum samples, a percentage inhibition (PI) value ≥ 50% was considered to indicate an immune response to FMDV Asia1. The VNT was performed according to the WOAH manual [8]. Two-fold serial dilutions of serum samples were prepared and then incubated with 100 TCID_50_/50 µL (50% tissue culture infective dose) of live virus at 37 °C for 1 h. Subsequently, LF-BK cells (2 × 10^4^ cells/well) were added and incubated at 37 °C in 5% CO_2_ for 3 days. The cytopathic effect was evaluated to determine the Ab titers calculated as the log_10_ of the reciprocal Ab dilution required to neutralize 100 TCID_50_ of the virus using the Spearman–Karber method [36,37].

### 2.8. NanoBiT-VNT Using HiBiT-AS Entry

**Figure 2:**
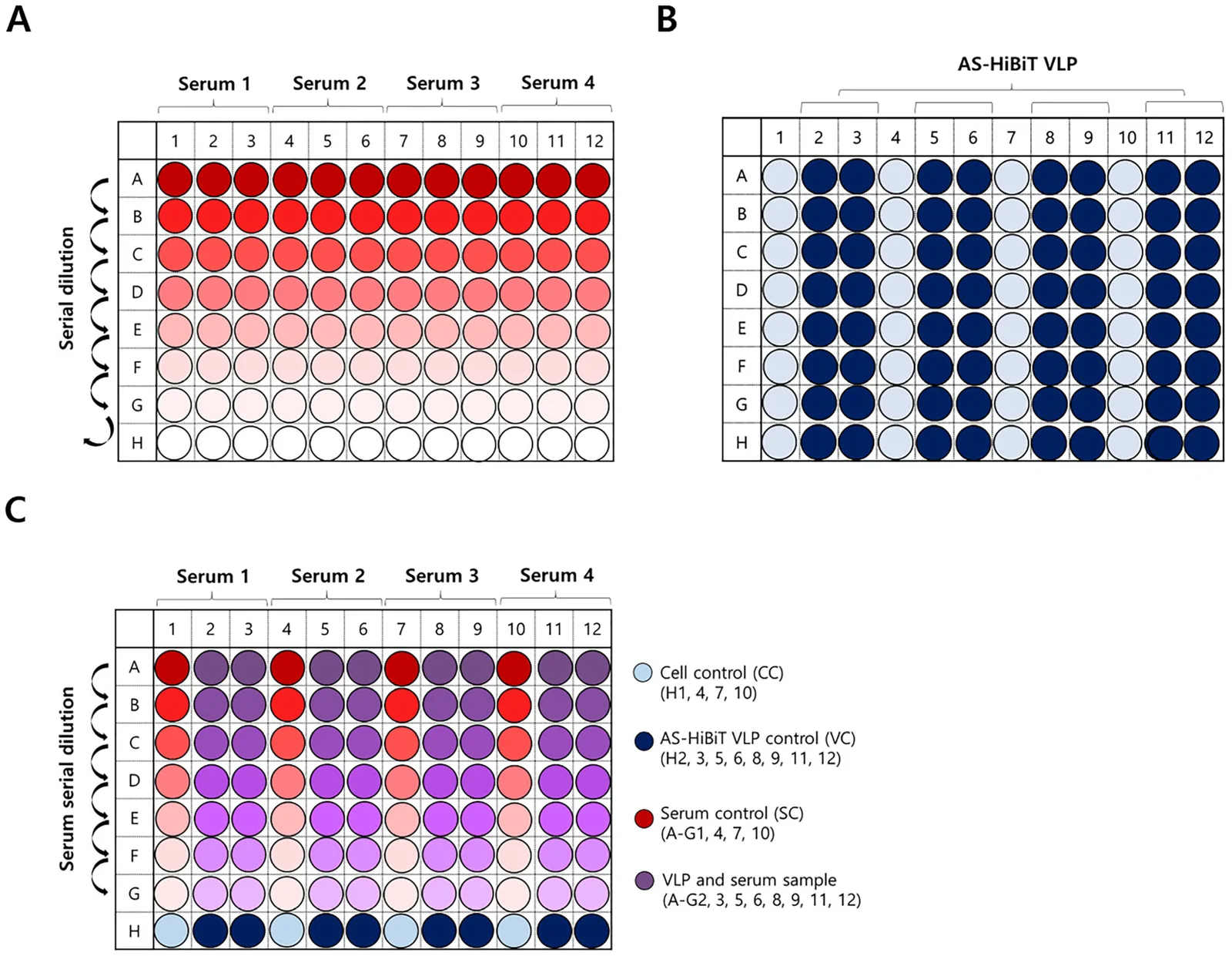
Layout of U-bottom 96-well plates for the NanoBit-based VNT using HiBiT-tagged AS VLPs. (**A**) Serial dilutions of different serum samples across rows A to G of a plate. Row H contains no serum as a control. (**B**) AS-HiBiT VLPs were added to columns 2, 3, 5, 6, 8, 9, 11, and 12. Serum controls were set in columns 1, 4, 7, and 10, and cell controls were set in row H. (**C**) The plate setup and results of the interaction between serum and AS-HiBiT VLPs. Red wells represent serum controls, blue wells indicate AS-HiBiT VLPs, and purple wells show the serum-AS-HiBiT VLP reactions.

NanoBiT-VNT was performed using stable LgBiT cells in a white 96-well plate, with 50 µL volumes for each step. Serum-free DMEM was added to all wells except A1–A12, where a 1/8 serum dilution was initiated, followed by two-fold serial dilutions across three rows per serum (Figure 2A). Previously titrated AS-HiBiT VLPs were added in columns 2, 3, 5, 6, 8, 9, 11, and 12 in rows A to H (row A–G: VLP/serum mixture, SC; row H: AS-HiBiT VLP control, VC), each 50 µL of AS-HiBiT VLP suspension was expected to contain approximately 1–2 × 10^4^ RLU of luciferase activity. Serum-free DMEM was added to columns 1, 4, 7, and 10 in rows A to H (row A–G: serum control, SC; row H: cell control, CC) (Figure 2B). It was incubated at 37 °C for 1 h, and the contents were transferred to a plate seeded with stable LgBiT cells that had been washed with PBS (Figure 2C). After incubation at 37 °C for 1 h, the plate was washed with PBS and filled with serum-free DMEM. Luciferase activity was detected using a Promega GloMAX luminometer and a Nano-Glo Luciferase Assay System (integration time: 0.8 s). The PI (%) of luciferase activity from each serum dilution was calculated as follows:

$$100-[\frac{mean\;RLU\;from\;each\;sample\;(serum\;and\;VLP)-average\;mean\;RLU\;from\;samples\;SC}{average\;mean\;RLU\;from\;VC\;samples-average\;mean\;RLU\;from\;CC\;samples}\times 100]$$

Antibody titers were determined from wells with a PI value of ≥ 50%, expressed as the log₁₀ of the reciprocal of the highest serum dilution where neutralizing antibodies (NAbs) were detected, following the Spearman–Karber method [36,37].

### 2.9. Statistical Analysis

All data are presented as means ± SEM. Individual variances were computed for each comparison, and statistical analyses were performed using one- or two-way ANOVA, followed by Tukey’s or Sidak’s multiple comparison tests using Prism software (version 8.4.3; GraphPad Prism Software, San Diego, CA, USA); *, *p* < 0.05; **, *p* < 0.01; ***, *p* < 0.001; ****, *p* < 0.0001; and ns, *p* > 0.05 (not significant).

## 3. Results

### 3.1. Transient Expression of LgBiT and AS-HiBiT in HEK293T Cells Restored Nano-Luc Activity

To assess whether the two components of split Nano-Luc (HiBiT and LgBiT) can interact and restore Nano-luc activity upon encountering each other, HEK293T cells were transfected with LgBiT, AS, or AS-HiBiT plasmids (Figure 1A). The protein expression was analyzed via Western blotting (Figure 1B). As expected, bands reactive to anti-HiBiT Abs were observed in samples transfected with AS-HiBiT alone or co-transfected with LgBiT and AS-HiBiT. Additionally, cells transfected with the LgBiT plasmid or co-transfected with LgBiT and AS-HiBiT displayed positive signals when detected with the anti-LgBiT Ab (Figure 1B). These results confirmed the functionality of the plasmids. Furthermore, we observed strong overexpression of luciferase activity (≥10^6^ RLU) in cells co-transfected with the LgBiT and AS-HiBiT plasmids, whereas the other transfected groups exhibited only background luciferase activity (≤10^2^ RLU values) (Figure 1C).

### 3.2. Production of AS-HiBiT VLPs

**Figure 3:**
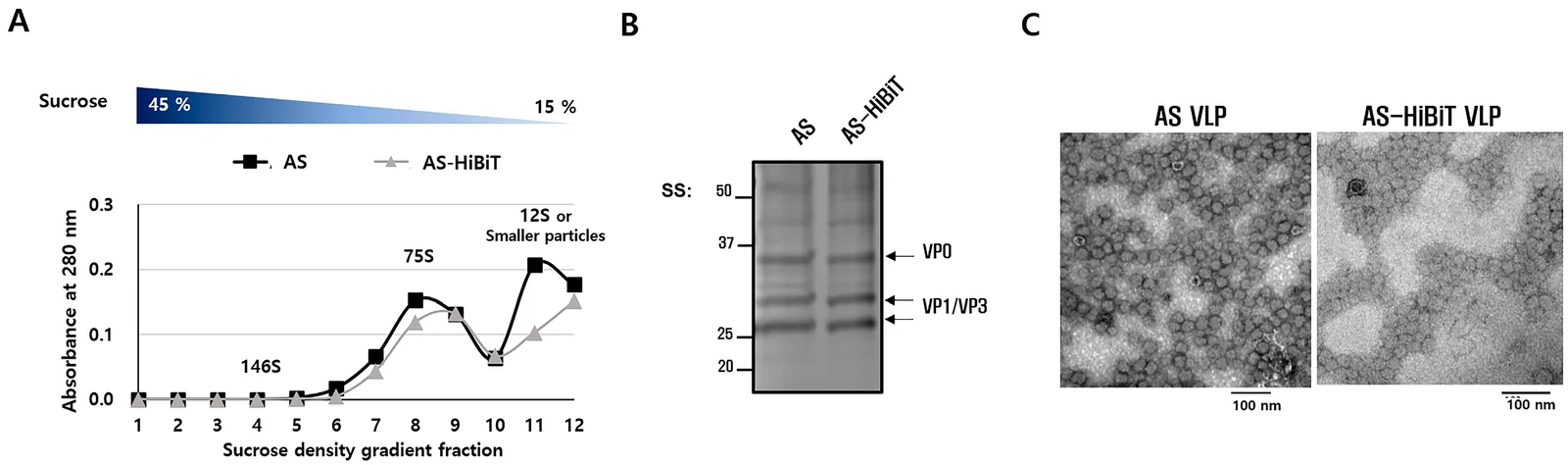
Production of AS and AS-HiBiT VLPs. (**A**) Sucrose density gradient ultra-centrifugation of AS and AS-HiBiT VLPs. Absorbance was measured at 280 nm, and fractions 6-9 corresponding to the 75S peak were collected for further analysis. (**B**) SDS-PAGE and Western blot analysis of the collected fractions. The silver-stained SDS-PAGE gel confirms the presence of AS and AS-HiBiT VLP proteins. (**C**) Transmission electron microscopy images of purified AS (left) and AS-HiBiT VLPs (right) showing the VLPs. Scale bar = 100 nm.

VLPs were concentrated and purified using sucrose density gradient centrifugation, with fractions 6–9 showing higher absorbance than others (Figure 3A). After concentrating and purifying these fractions, silver staining and Western blotting were performed to confirm the presence of VLP proteins (Figure 3B). These assays revealed distinct bands corresponding to the VLP proteins, indicating that the viral proteins were successfully concentrated and purified from the selected fractions (Figure 3B). TEM analysis was conducted to further confirm the structural characteristics of AS VLPs and AS-HiBiT VLPs (Figure 3C). The TEM images clearly showed VLPs measuring 25–30 nm in diameter with a regular arrangement of size and morphology. These findings confirmed the successful purification of VLPs and HiBiT-tagged VLPs.

### 3.3. Optimization of AS-HiBiT VLP Entry Conditions and Luciferase Activity in Stable LgBiT Cells

**Figure 4:**
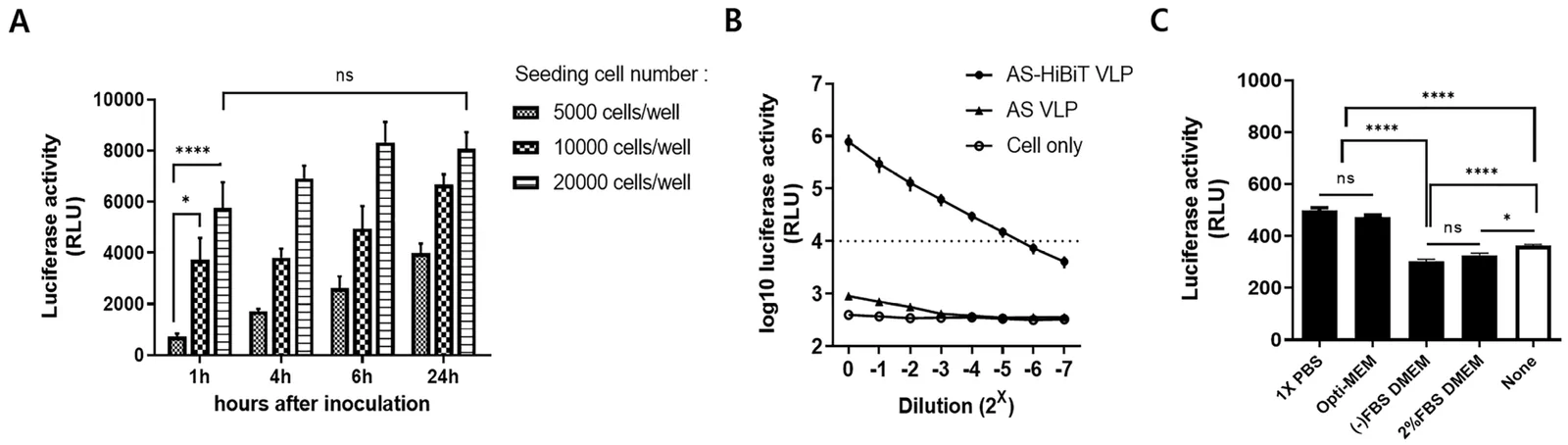
AS-HiBiT VLP binding and entry into stable LgBiT-expressing cells. (**A**) The effect of AS-HiBiT VLP incubation time in stable cells seeded at 5000, 10,000, and 20,000 cells/well at 37 °C, with measurements taken at multiple time points up to 24 h. The dotted line represents the detection threshold of 10^4^ RLU for luminescence activity. (**B**,**C**) The effect of AS-HiBiT VLP dilution and medium conditions on luciferase activity in stable LgBiT cells (20,000 cells/well) at 37 °C for 1h. All data represent the means ± SEM of triplicate wells. Statistical significance was analyzed using two-way ANOVA followed by Tukey’s multiple comparisons test. *, *p* < 0.05; ****, *p* < 0.0001; and ns, *p* > 0.05.

To determine the optimal conditions for AS-HiBiT VLP entry into stable LgBiT cells, luciferase activity was measured based on cell number and incubation time (Figure 4A). Luciferase activity significantly increased with cell number, identifying 2 × 10⁴ cells/well as optimal. No significant differences in luciferase activity were observed between a 1 h incubation and longer times at this cell density, suggesting that 2 × 10⁴ cells/well and a 1 h incubation were optimal. Luciferase activity also decreased progressively with lower AS-HiBiT VLP concentrations (Figure 4B). Wells containing AS VLPs without HiBiT or cells alone showed RLU values below 1 × 10^3^, whereas a minimal luciferase activity of 10⁴ RLU was needed for the neutralization assay. Furthermore, luciferase activity varied with different cell culture media (Figure 4C), showing higher values in PBS and Opti-MEM compared with serum-free DMEM, which produced the lowest RLU values.

### 3.4. Application of NanoBiT Assay Using AS-HiBiT VLP with Guinea Pig Serum

**Figure 5:**
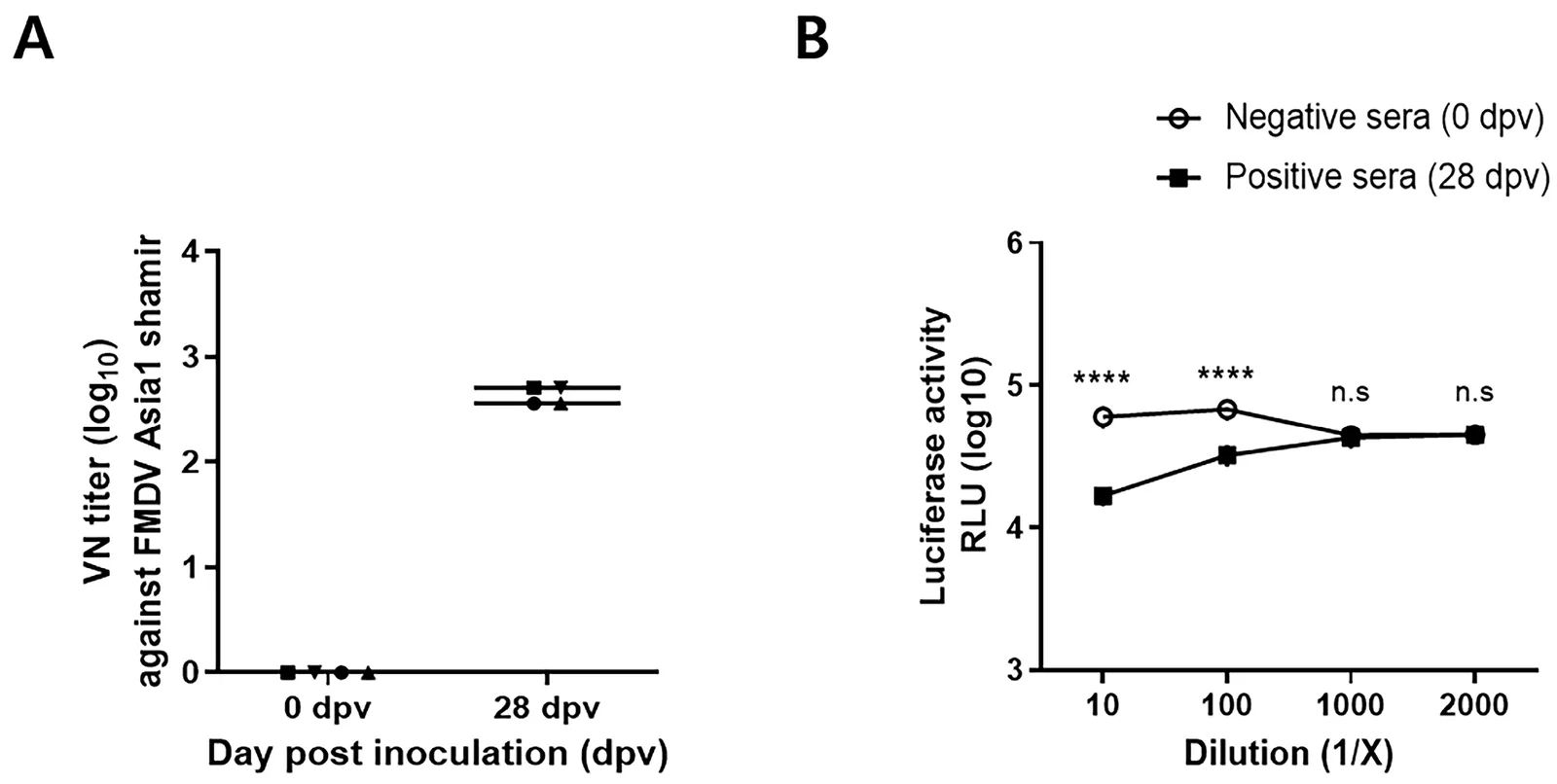
Application of the AS-HiBiT VLPs with guinea pig sera for NanoBiT assay. (**A**) Sera from guinea pigs (*n* = 4) vaccinated with a commercial FMD vaccine (including AS) were tested to measure the neutralizing antibody titers of FMD AS using the traditional VNT. The sera collected at 28 dpv showed high neutralizing antibody titers against AS and were classified into the positive group, whereas the sera collected at 0 dpv did not show neutralizing antibody titers and were classified into the negative group. (**B**) The negative and positive sera were applied to the NanoBiT assay. All data represent means ± SEM analyzed using two-way ANOVA following Sidak’s multiple comparisons test. ****, *p* < 0.0001; ns, no significant difference.

To evaluate the applicability of AS-HiBiT VLP and stable LgBiT cells in the NanoBiT assay, AS-HiBiT VLPs were reacted with guinea pig serum and applied to the cells. Using the traditional VNT with live AS revealed a clear distinction between negative (0 dpv) and positive (28 dpv) serum. Positive sera showed a VN titer greater than 1:100, whereas negative sera showed no detectable titers (Figure 5A). Negative and positive sera were then diluted 10-, 100-, 1000-, and 2000-fold and subjected to the optimized NanoBiT assay conditions (Figure 5B). The negative serum maintained consistent luciferase activity regardless of the dilution factor, whereas the positive serum exhibited a significant difference compared with the negative serum in the 10- and 100-fold dilution groups. However, at dilutions of 1000-fold or higher, the luciferase activity of the positive serum was similar to that of the negative serum.

### 3.5. NanoBiT-VNT Assay to Detect NAbs in Pigs

**Figure 6:**
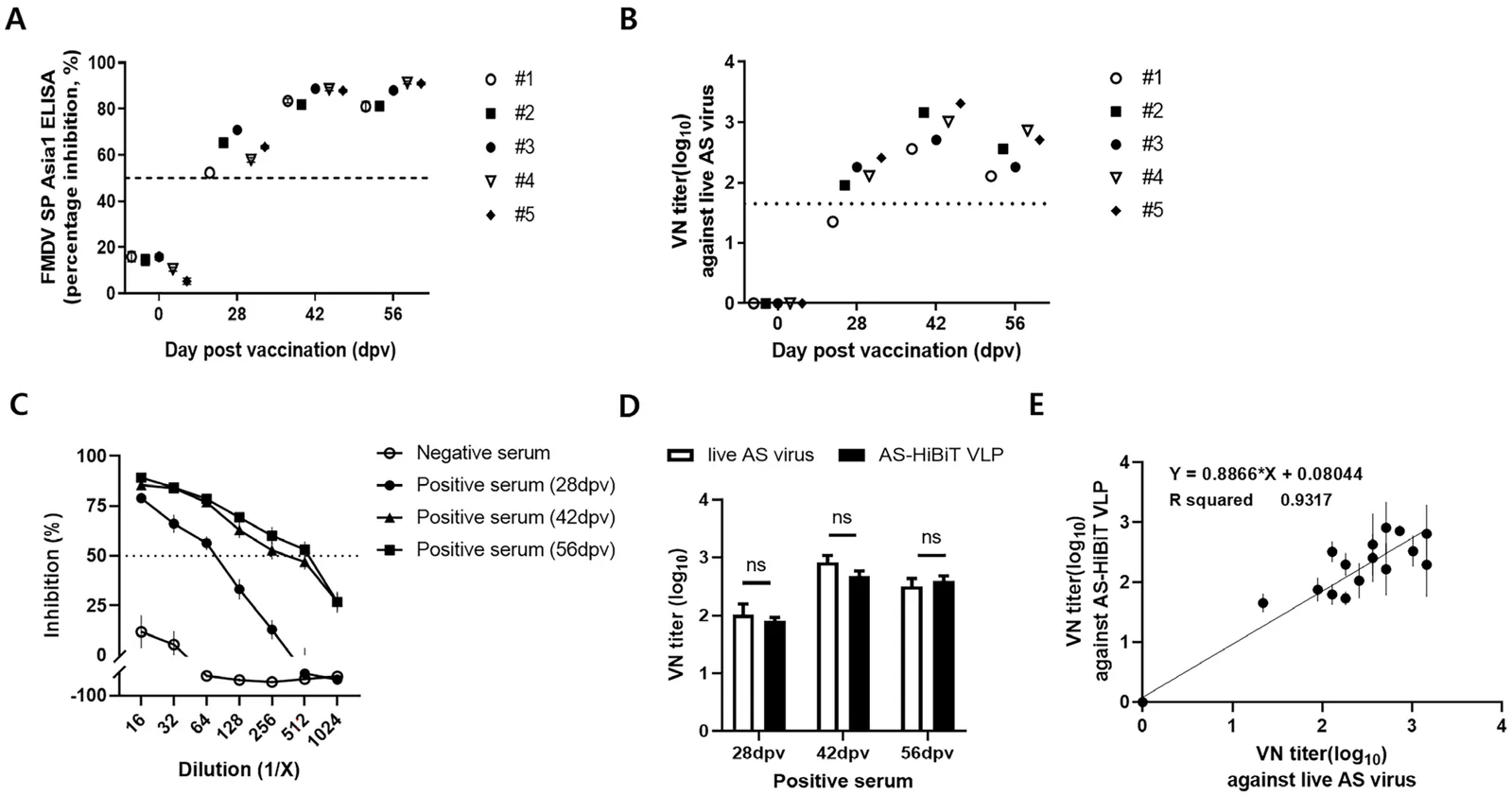
Time-dependent neutralizing antibody responses after vaccination and correlation between traditional VNT and NanoBiT-VNT in pig sera. (**A**) FMDV SP Asia1 ELISA results for five pigs collected at 0, 28, 42, and 56 dpv. The PI (%) gradually increased after vaccination, indicating the induction of antibody responses over time. (**B**) VN titers against live AS virus for five pigs measured at 0, 28, 42, and 56 dpv. A clear increase in VN titers was observed, especially after 28 dpv. (**C**) Inhibition (%) of AS-HiBiT VLPs using negative and positive sera in stable LgBiT cells analyzed as indicated in Section 2.8. The serum samples were diluted, starting from 1:16 to 1:1024. Positive sera showed higher inhibition (%) at various serum dilutions, indicating the presence of neutralizing antibodies. (**D**) Comparison of VN titers (log₁₀) between the traditional VNT and NanoBiT-VNT for sera collected at 28, 42, and 56 dpv. No significant differences were observed between the two methods, suggesting comparable accuracy. Statistical significance was analyzed using two-way ANOVA, followed by Sidak’s multiple comparisons test. N.s., no significant difference. (**E**) Correlation analysis between VN titers obtained using the traditional VNT and NanoBiT-VNT. A strong correlation was observed (R^2^ = 0.9317), demonstrating the consistency between the two methods. Samples were analyzed in duplicates, and data are shown as means ± SEM.

We evaluated the applicability of the NanoBiT-VNT using pig sera. SP ELISA and the traditional VNT results showed a gradual increase in PI (%) and VN titers, especially indicating successful NAb formation (Figure 6A,B). Using AS-HiBiT VLP, NanoBiT-VNT detected NAb titers by converting luciferase activity (RLU) into PI (%) values (Figure 6C). Negative sera (0 dpv) exhibited PI values < 50%. In contrast, positive sera collected at 28, 42, and 56 dpv demonstrated PI (%) values above 50% at serum dilutions ranging from 1:16 to 1:64. Specifically, the 28 dpv positive sera showed PI (%) < 50% at 1:128 dilution, whereas the 42 and 56 dpv positive sera showed PI (%) < 50% only at dilutions of 1:512 or higher (Figure 6C). Furthermore, the VN titers measured through PI (%) in the NanoBiT-VNT were consistent with those measured through the traditional VNT (Figure 6D). The VN titers from the NanoBiT-VNT and the traditional VNT (Figure 6E) showed a high correlation (R² = 0.9317). This finding indicates that the HiBiT VLP-based NanoBiT assay is a sensitive method for detecting NAbs in pig sera.

### 3.6. Potential Application of NanoBiT-VNT for Enhanced NAb Detection

**Figure 7:**
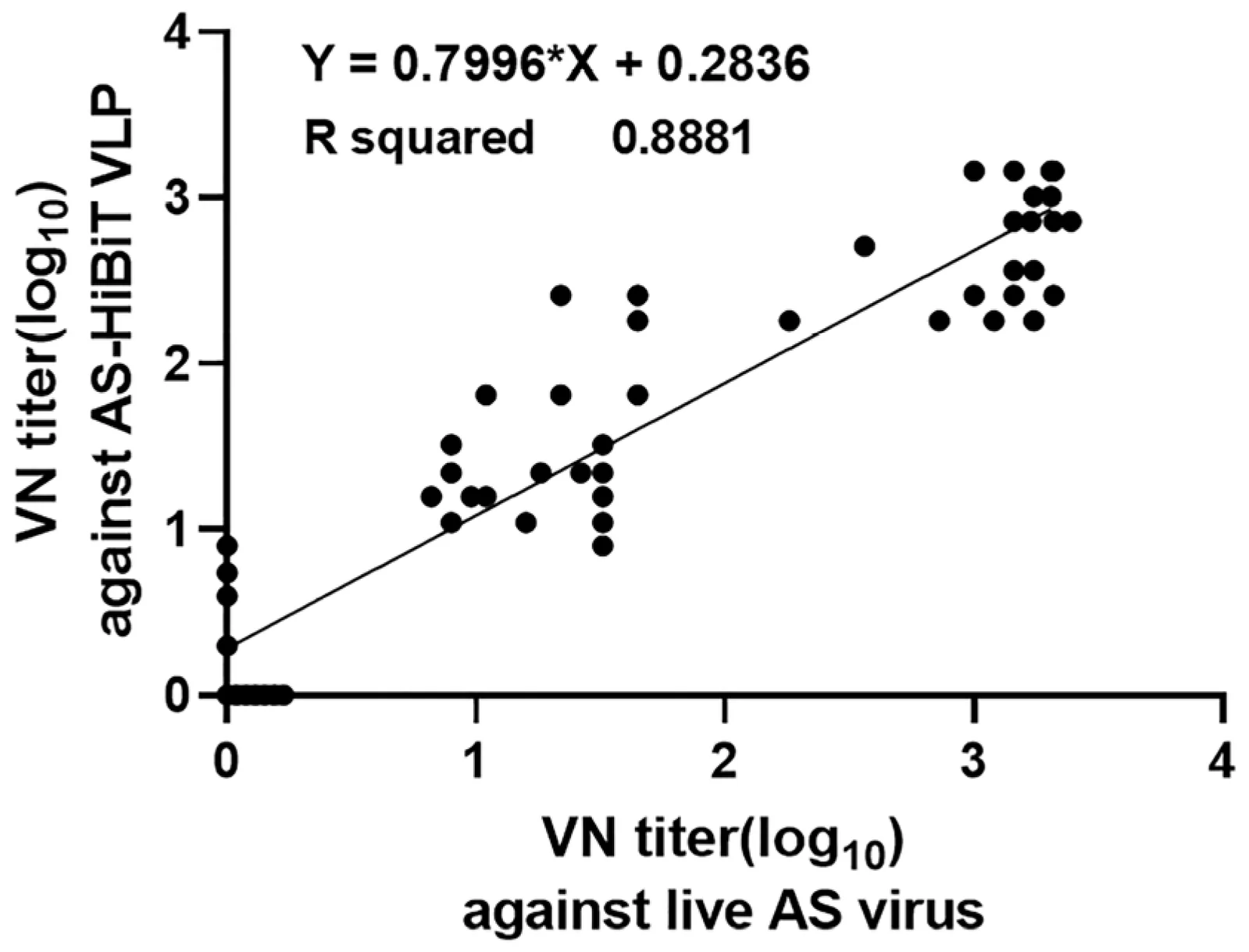
Comparison of VNTs between live FMD AS and AS-HiBiT VLPs in various anti-AS pig sera (*n* = 58). VN titers (log_10_) against FMDV AS were measured using both the traditional VNT and the AS-HiBiT VLP-based NanoBiT-VNT in pig sera. Correlation analysis between VN titers measured through the traditional VNT and the AS-HiBiT VLP-based NanoBiT-VNT. The regression line indicates a strong positive correlation (R^2^ = 0.8881), further supporting the consistency of the two methods.

To further validate the assay, we compared NAb titers between the traditional VNT and NanoBiT-VNT using AS-HiBiT VLPs with a diverse set of anti-AS pig sera (*n* = 58). This suggests that the NanoBiT-VNT based on AS-HiBiT VLPs exhibits accuracy comparable to that of the traditional VNT and is capable of sensitively detecting NAbs in pig sera. Furthermore, correlation analysis between the two assays demonstrated high values of R² = 0.881. This strong correlation indicates that the NanoBiT-VNT provides highly consistent NAb titers with those obtained from the traditional VNT, supporting its potential as a reliable alternative method for measuring Nabs (Figure 7).

## 4. Discussion

We developed an AS-HiBiT VLP-based NanoBiT assay as a substitute for the VNT in FMD research. VN titers measured in pig sera showed a high correlation with the traditional gold standard VNT, with no significant differences, indicating that the NanoBiT assay detects NAbs with comparable accuracy and sensitivity. Unlike the traditional VNT, the AS-HiBiT VLP-based NanoBiT assay eliminates the need for live FMDV and is compatible with BL-2 facilities, enhancing biosafety and simplifying experimental procedures. This suggests that the NanoBiT assay is a faster and safer method for measuring NAbs.

According to the WOAH, FMD occurs in multiple regions, including Africa, Asia, the Middle East, and South America [38]. Despite vaccination efforts in Africa, rates remain low due to financial and infrastructure limitations, including a lack of refrigeration and BL-3 facilities. The AS-HiBiT VLP-based NanoBiT assay offers a safer, more efficient alternative for BL-2 facilities, allowing cost-effective vaccine evaluation and sensitive monitoring of NAbs, even in underdeveloped countries with limited infrastructure.

We recently reported that the NanoBiT assay can be applied to FMD research by tagging inactivated FMDV with HiBiT and reacting it with stable LgBiT cells [15]. Furthermore, measuring NAbs using the AS-HiBiT VLP-based NanoBiT assay is a novel approach introduced for the first time in FMD research. The insertion of HiBiT at 153S in VP1 was designed based on its structural flexibility and its proximity to the RGD motif, a key determinant of integrin binding, which ensures that the modification does not interfere with viral function [16]. Our findings confirm that this insertion site is well tolerated and enables effective neutralizing antibody detection using the NanoBiT-VNT assay. While previous studies have successfully utilized FMDV VLPs for serological detection using ELISAs [39,40], our study demonstrates an innovative application of VLPs in a bioluminescence-based neutralization test, offering higher sensitivity while maintaining the capability to be conducted in BL-2 facilities, providing a safer and more efficient alternative for neutralizing antibody detection. This method enables the measurement of VN titers at various serum dilutions after vaccination and serves as an important indicator of vaccine efficacy monitoring.

To evaluate the applicability of the AS-HiBiT VLP-based NanoBiT assay in animal models, we first conducted experiments using guinea pig sera before applying this method to pig sera. Unlike the VNT assay, which determines precise titers through two-fold serial dilutions, the NanoBiT assay used in this study was performed with ten-fold serial dilutions to assess its feasibility. The results showed that the VNT detected a 320-fold difference in antibody titers between positive and negative sera, whereas, in the NanoBiT assay, the distinction between positive and negative sera was not apparent at dilutions between 100-fold (log2) and 1000-fold (log3). These findings indicate that while the NanoBiT assay offers a high-throughput and biosafe alternative to the VNT, the narrower differentiation window may pose limitations in distinguishing low neutralizing antibody (nAb) titers from negative samples. Subsequently, we compared both assays using pig sera with varying neutralizing antibody titers.

The AS-HiBiT VLP-based NanoBiT assay provides a safe and effective method for detecting neutralizing antibodies against FMDV. By eliminating the need for live virus handling, it offers a practical alternative to the conventional VNT, reducing reliance on BL-3 facilities and improving accessibility for laboratories with limited high-containment resources. However, the structural stability of VLPs may affect their ability to enter cells. Previous reports have indicated a significant reduction in cell entry of human NoV VLP-HiBiT after heat treatment at temperatures above 65 °C [41,42]. This may be due to the disruption and/or denaturation of the capsid, which reduces the ability of the VLPs to bind to cell surface receptors. Similarly, structural disruption and/or modification of AS-HiBiT VLPs could potentially reduce cell entry efficiency. Although AS-HiBiT VLPs demonstrated a strong correlation with the conventional VNT results, structural differences between VLPs and authentic viral particles may influence antigenicity. To further validate this method, future studies should investigate how these structural differences impact antibody recognition, particularly in relation to HiBiT insertion within the VP1 GH-loop. Additionally, ensuring the structural stability of AS-HiBiT VLPs will be crucial for maintaining reproducibility and accuracy in serological assays.

The AS-HiBiT VLP-based NanoBiT assay developed in this study offers a sensitive and practical alternative for FMDV serological testing. Unlike conventional virus neutralization tests (VNTs), which require live virus handling in BL-3 facilities, this assay eliminates the need for virus cultivation while maintaining a strong correlation (R² = 0.8881) with traditional VNT methods. Enabling testing under BL-2 conditions provides a more accessible and cost-effective approach to neutralizing antibody detection, reducing reliance on BL-3 facilities. These advantages suggest that this method could be extended beyond FMDV research. It can also be applied to measure NAb responses against other animal infectious disease viruses, providing a tool for the rapid evaluation of vaccine efficacy. In future studies, the efficacy of the AS-HiBiT VLP-based NanoBiT assay should be evaluated using other animal models to expand its applicability. Furthermore, this method should be applied to measure NAbs against various FMDV serotypes and to validate its effectiveness. The AS-HiBiT VLP-based NanoBiT assay provides a rapid, safe, and effective alternative for FMDV neutralizing antibody detection, allowing for high-throughput screening in BL-2 laboratories.

## Data Availability

The original contributions presented in this study are included in the article. Further inquiries can be directed to the corresponding authors.
